# Modulation of psoriatic-like skin inflammation by traditional Indian medicine Divya-Kayakalp-Vati and Oil through attenuation of pro-inflammatory cytokines

**DOI:** 10.1016/j.jtcme.2021.09.003

**Published:** 2021-09-20

**Authors:** Acharya Balkrishna, Sachin Sakat, Kheemraj Joshi, Rani Singh, Sudeep Verma, Pardeep Nain, Kunal Bhattacharya, Anurag Varshney

**Affiliations:** a Drug Discovery and Development Division, Patanjali Research Institute, NH-58, Haridwar, 249405, Uttarakhand, India; b Department of Allied and Applied Sciences, University of Patanjali, Patanjali Yog Peeth, Haridwar, India

**Keywords:** Divya-kayakalp-vati, Divya-kayakalp-oil, Skin psoriasis, Inflammation, Cytokine release

## Abstract

**Background and aim:**

Psoriasis (Ps) is a chronic skin inflammatory disorder, that progresses to scaly-red dermal plaque formations associated with inflammation. Divya-Kayakalp-Vati (DKV) and Divya-Kayakalp-Oil (DKO) are traditional Ayurveda herbo-mineral formulations, that are prescribed for the treatment of inflammatory dermal ailments. In the present study, we evaluated the efficacy of Divya-Kayakalp-Vati and Divya-Kayakalp-Oil (DKV-O) combined treatment in ameliorating Ps-like skin inflammation under *in-vitro* and *in-vivo* conditions.

**Experimental procedure:**

Efficacy of DKV-O was analyzed in λ-carrageenan-treated Wistar rats paw edema and 12-O-tetradecanoylphorbol 13-acetate (TPA)-treated CD-1 mouse ear edema models through physiological and histopathological analysis. Mode of action for the DKV-O was studied in LPS-stimulated THP-1 cells through pro-inflammatory cytokine analysis. Observed effects were correlated to the phytochemicals constituents of DKV-O analyzed using the HPLC method.

**Result and conclusion:**

DKV and DKO formulations were individually found to contain phytochemicals Gallic acid, Catechin, Berberine, Curcumin, Phenol and Benzoic acid. DKV-O treatment significantly reduced the paw volume and edema in Wistar rats stimulated through λ-carrageenan-treatment. Furthermore, DKV-O treatment significantly reduced the ear edema and enhanced biopsy weight, epidermal thickness, inflammatory lesions and influx of neutrophils stimulated by TPA-treatment in CD-1 mice. DKV alone ameliorated the LPS-stimulated release of Interleukin (IL)-6, IL-17A, IL-23, and Tumor Necrosis Factor-alpha cytokines in the THP-1 cells.

Taken together, DKV-O showed good efficacy in ameliorating acute systemic inflammation stimulated by effectors such as, λ-carrageenan and TPA in animal models. Hence, Divya-Kayakalp-Vati and Divya-Kayakalp-Oil co-treatment can be further explored as an anti-inflammatory treatment against dermal diseases like psoriasis and atopic dermatitis.

## Highlight of Findings and Novelities

• Divya-Kayakalp-Vati (DKV) and Divya-Kayakalp-Oil (DKO) are Indian herbo-mineral Ayurveda anti-inflammatory formulations.
• Combined treatment of DKV and DKO (DKV-O) ameliorated λ-Carrageenan stimulated paw-edema and -volume increase in Wistar rats.
• DKV-O treatment eliminated 12-O-tetradecanoylphorbol 13-acetate stimulated ear-edema, and inflammation in CD-1 mouse ear.
• DKV reduced the bacterial lipopolysaccharide (LPS)-stimulated release of pro-inflammatory cytokine release in THP-1 cells.
• Finally, DKV-O reduced chemically stimulated psoriasis-like inflammation by means of its anti-inflammatory phytochemicals.

## Graphical abstract

**Figure:**
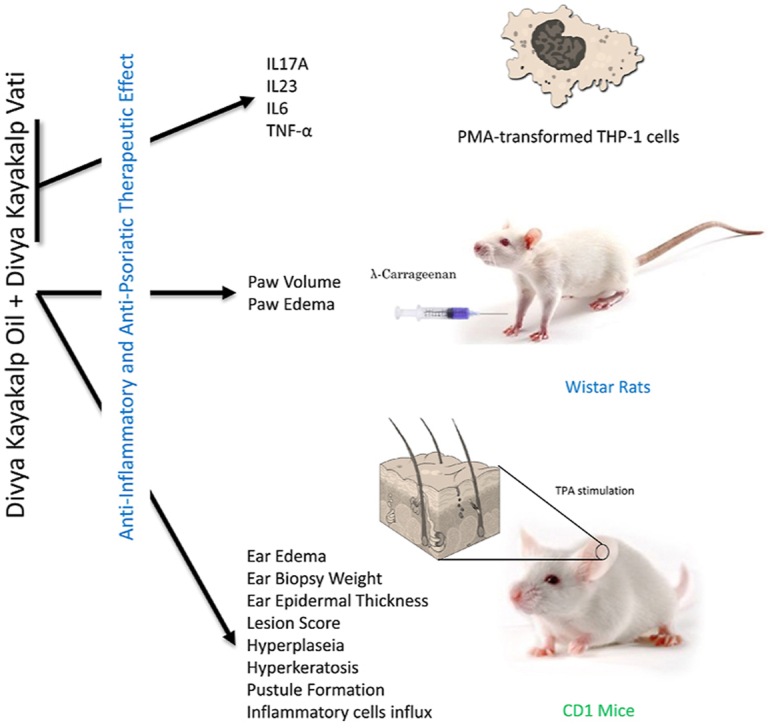

## Abbreviations

ANOVA: One-way Analysis of Variance
CPCSEA: Committee for the Purpose of Control and Supervision of Experiments on Animals
DEXA: Dexamethasone
DKO: Divya-Kayakalp-Oil
DKV: Divya-Kayakalp-Vati
DKV-O: Divya-Kayakalp-Vati and Divya-Kayakalp-Oil co-treatment
ELISA: Enzyme-Linked Immunosorbent Assay
HPLC: High-Performance Liquid Chromatography
IAEC: Institutional Animal Ethical Committee
ICH: International Conference on Harmonization
IL-17A: Interleukin 17A
IL-23: Interleukin 23
IL-6: Interleukin-6
INDO: Indomethacin
LPS: lipopolysaccharide
Na-CMC: Sodium-carboxymethyl cellulose
NC: Normal Control
PPIA: Peptidyl-prolyl cis-trans isomerase (PPIase) family
Ps: Psoriasis
RT-PCR: Real-time polymerase chain reaction
TNF-**α**: Tumor necrosis factor-alpha
TPA: 12-O-tetradecanoylphorbol 13-acetate

## 1 Introduction

Psoriasis (Ps) is an innate and adaptive autoimmune disorder, which affects approximately 125 million people worldwide.^1^ Ps is a systemic inflammatory disease represented by the activation of the innate and adaptive immune systems and increased release of pro-inflammatory cytokines.^2^, ^3^, ^4^, ^5^, ^6^ Onset of the disease can cause long-term damage to multiple tissues and organs. Dermal Ps cause a variety of lesion phenotypes with Ps vulgaris being the common manifestation represented by the occurrence of scaly-red inflammatory plaques having enhanced hyperkeratosis, parakeratosis and reduced stratum granulosum, and pain with tenderness.^7^^,^^8^ Currently, there is no permanent cure available for Ps; and anti-inflammatory drugs are applied to quell the Ps flaring.

Recently, herbal formulations have gained popularity as ‘cosmeceuticals’ as they don't produce any side effects.^9^ Divya-Kayakalp-Vati (DKV) and Divya-Kayakalp-Oil (DKO) have been formulated following method prescribed in ancient India Ayurvedic textbooks *Bhavprakash Nighantu* and *Ayurved Saar Sangrah* for treatment of dermal etiologies.^10^^,^^11^ DKV is composed of herbo-mineral components- Panvad (*Cassia tora* L.), Haldi (*Curcuma longa* L.), Daru Haldi (*Berberis aristata* DC.), Khair (*Acacia catechu* (L.F.) Willd.), Karanj (*Caesalpinia bonducella* (L.) Fleming), Neem (*Azadirachta indica* A. Juss.), Amla (*Emblica officinalis* Gaertn.), Manjishta (*Rubia cordifolia* L.), Giloy (*Tinospora cordifolia* (Willd.) Hook. f. & Thomson), Chirayata (*Swertia chirata* Buch. -Ham. (ex. Wall)), Kutaki (*Picrorhiza kurroa* Royale ex. Benth.), Dronpushapi (*Leucas cephalotes* (Roth) Spreng.), Satyanashi (*Argemone Mexicana* L.), and Rasmanikya (Red Sapphire ash) (Suppl. Table 1). Several of the phytocomponents have been reported to display anti-inflammatory activities.^12^, ^13^, ^14^, ^15^, ^16^, ^17^, ^18^, ^19^, ^20^

Animal models for λ-carrageenan-stimulated Wistar rat paw edema and 12-O-tetradecanoylphorbol 13-acetate (TPA)-stimulated CD-1 mouse ear edema represents acute systemic inflammation that is similar to Ps-like morbidities through the production of cytokine, reactive oxygen and nitrogen species.^21^^,^^22^ λ-carrageenan-treatment induces a bi-phasic inflammatory response, with the early response represented by an increase in vascular permeability, and the later-stage represented through an induction of edema.^23^ TPA treatment stimulates the development of dermal inflammation including IL17/IL23 axis, edema, epidermal hyperplasia and has been used for studying the Ps-like disease-modulating efficacy of different medicines.^24^, ^25^, ^26^, ^27^, ^28^

In the present study, using λ-carrageenan-stimulated Wistar rats paw-edema and TPA-stimulated CD-1 mice ear edema models, we analyzed the anti-inflammatory activity for combined treatment of DKV (oral) and DKO (topical) (DKV-O) in ameliorating Ps-like inflammation. We investigated the anti-inflammatory mode of action for DKV through modulation of cytokine release in bacterial lipopolysaccharide (LPS)-stimulated phorbol 12-myristate-13-acetate (PMA)-transformed THP-1 cells. We summed up this study with a chemical analysis of DKV and DKO; and appended those findings with observed biological efficacies.

## 2 Materials and methods

### 2.1 Chemicals and reagents used

DKV and DKO were procured from Divya Pharmacy, Haridwar, India. RPMI-1640 cell culture media, FBS, antibiotic/antimycotic were purchased from Gibco, USA. Bacterial origin endotoxin LPS (O111:B4), λ-carrageenan, TPA, indomethacin (INDO), DEXA, and standard compounds (>95% by HPLC) such as Gallic acid, Catechin, Berberine, Phenol, Benzoic acid, and Curcumin were purchased from Sigma-Aldrich (St. Louis, MO, USA). ELISA kits for TNF-α and IL-6 were purchased from BD Biosciences, USA. Primers for the study of IL-17A and IL-23 were purchased from Eurofins, India. HPLC grade acetonitrile, *ortho*-phosphoric acid, and diethylamine along with hematoxylin, potassium aluminum sulfate dodecahydrate, and mercury (II) oxide red were purchased from Merck India Pvt. Ltd, India. Eosin yellow and ferric chloride were purchased from HiMedia Laboratories, India. All other chemicals and reagents purchased were of the highest commercial grade.

### 2.2 Herbal sample preparation

20 g of DKV (Batch no. A-KKVE090) was pulverized and refluxed for 6 h at 88 °C in 300 mL of 70% ethanol. The solution was filtered and dried at 45 °C through evaporation under vacuum in a rotary film evaporator and stored until further use.

90 g of DKO (Batch no. BKKT056) was mixed in 200 mL of 90% methanol and stirred for 1 h. The prepared solution was stored at −20 °C for 2 days. Following, separation of methanol from the frozen oil layer, it was removed manually. DKO was thawed, filtered, and dried using a Rotavapor. The dried DKO residue was resuspended in 90% methanol, and stored for further experiments.

### 2.3 High-performance-liquid-chromatography (HPLC) analysis

Chemical analysis of the DKV and DKO was performed using the binary HPLC system (Waters Corporation, Milford, MA, USA). Clear separations of DKV and DKO were done using an aqueous 0.1% *ortho*-phosphoric acid (v/v) (pH 2.5), diethylamine, and acetonitrile mobile-phase. A reversed-phase C18 analytical column of 4.60 × 250 mm and 5 μm particle sizes (Sunfire, Waters, USA) set at 35 °C was utilized for the analysis of Gallic acid, Phenol, Benzoic acid, and Catechin at 275 nm, and Berberine and Curcumin at 475 nm. A gradient-based program was used for the analysis and was repeated six times to confirm repeatability (relative standard deviation of <2.5%).

### 2.4 Experimental animals

Male Wistar rats (8–10 weeks) and CD-1 mice (6–8 weeks) were procured from Liveon Biolabs Pvt. Ltd, India, and from Hylasco Biotechnology Pvt. Ltd, India (licensed supplier for Charles River Laboratory), respectively. Animals were placed under a controlled environment with a relative humidity of 60–70% and 12:12 h light and dark cycle in a registered animal house of Patanjali Research Institute, India (1964/PO/RC/S/17/CPCSEA). They were fed a standard pellet diet (Golden Feed, India) and sterile filtered water *ad libitum*. The study protocols were approved by the Institutional Animal Ethical Committee (IAEC) of Patanjali Research Institute vide approval numbers: PRIAS/LAF/IAEC-008 and PRIAS/LAF/IAEC-022 and implementation of the study were performed following the UK Animals (Scientific Procedures) Act, 1986 and EU Directive 2010/63/EU guidelines and regulations for animal experimentation.^29^

### 2.5 Evaluation of *in-Vivo* anti-inflammatory and anti-Ps like efficacies

#### 2.5.1 λ-carrageenan-stimulated paw-edema Wistar rat model

λ-carrageenan-stimulated paw-edema test was performed following the procedure mentioned earlier.^30^ Briefly, male Wistar rats were randomly divided into eight animals per group for studying the parameter basal paw volume using the Plethysmometer (Ugo Basile, Italy). Inflammation was stimulated by the subcutaneous injection of 0.1 ml λ-carrageenan (1% solution prepared in normal saline) into the plantar side of the left hind paw. The paw was marked with ink at the level of the lateral malleolus and the volume was measured every hour up to 5 h. Animals were treated with 75 mg/kg of DKV (p.o.) (1000 mg/day human equivalent dose) + 40 μl of DKO (T.A.), or, with 10 mg/kg of INDO (positive control) (p.o.), 1 h before the λ-carrageenan challenge. Vehicle control animals were treated with normal saline only. The onset of paw-edema and anti-inflammatory activity (%) was calculated for each animal/h using the formula:

(1) $$\left(\frac{\left[Mean\;paw\;edema\;of\;control\;animals-Mean\;paw\;edema\;of\;each\;test\;animals\right]}{Mean\;paw\;edema\;of\;control\;animals}\right)X\;100$$

#### 2.5.2 TPA-stimulated Ps-like skin inflammation CD-1 mouse model

The inhibitory efficacy of DKV-O in ameliorating Ps-like etiologies was studied in the TPA-stimulated CD-1 mouse model as defined earlier.^30^ Briefly, 20 μL of TPA solution prepared in acetone was applied topically on the right ear of CD-1 mouse at the concentration of 2.5 μg/ear every second day till the 10th day. The left ear was reserved as vehicle control and treated with 20 μL of acetone alone during the study duration. Ear thickness was measured every day using a digital Vernier caliper (Mitutoyo, Tokyo, Japan) and changes were determined by subtracting the ear thickness of day 0 from the respective time points. Based on treatments, animal groups were divided as normal control, vehicle control, TPA only (disease control), 0.2 mg/kg of DEXA (T.A.), and 150 mg/kg of DKV (p.o.) + 20 μl of DKO (T.A.).

### 2.6 Histopathological analysis

CD-1 mice were humanely sacrificed on day 10, exactly 6 h after the last drug treatment. Ear biopsy samples were fixed in 10% formalin and embedded in paraffin. The paraffin-embedded samples were sectioned using microtome to 5 μm thickness. The tissue samples were stained with hematoxylin and eosin dyes. Epidermal thickness (from the basal layer to stratum corneum) of the ear tissue sample was measured using Magcam DC5 microscopic camera (Magnus Opto Systems India Pvt. Ltd., Noida, India) equipped with a stage micrometer and was analyzed using MagVision image analysis software (Magnus Opto Systems India Pvt. Ltd., Noida, India). Distribution of the lesions was recorded as focal, multifocal, and diffused. Severity of the observed lesions and and neutrophil influx was recorded as NAD= No abnormality detected, 1 = minimal (<1%), 2 = mild (1–25%), 3 = moderate (26–50%), 4 = moderately severe/marked (51–75%), and 5 = severe (76–100%). Other parameters such as the extent of lesions, severity of hyperkeratosis, number and size of pustules, epidermal hyperplasia (measured in the interfollicular epidermis), the severity of inflammation in the dermis and soft tissue, and any other lesion(s) were also recorded and scored.

### 2.7 Cell culture of human monocyte (THP-1) cells

THP-1 cells were obtained from the National Centre for Cell Science, Pune, India. THP-1 cells were cultured in RPMI-1640 media, supplemented with 10% heat-inactivated fetal bovine serum (FBS), 1% penicillin-streptomycin (100 U/mL), 1% sodium pyruvate (1 mM), and 1% l-glutamine (4 mM); and were maintained in a humid and sterile environment at 37 °C and 5% CO_2_.

### 2.8 Real-time Polymerase Chain Reaction (RT-PCR) assay

THP-1 cells were seeded in 24 wells culture plates at the concentration of 5 x 10^5^ cells/well and stimulated with 20 ng/ml of PMA overnight. The next day, old media containing PMA was replaced and the transformed THP-1 cells were washed with lukewarm sterile PBS. THP-1 cells were replenished with fresh culture media and incubated for one day for stabilization. PMA-transformed THP-1 cells were pre-exposed to varying concentrations of DKV for a period of 1 h. After incubation time was over, the THP-1 cells were stimulated with LPS (1 μg/ml) for 24 h following completion of exposure time, cell culture media was separated and stored at −80 °C till further use. Cells were washed and total RNA was isolated using RNeasy mini kit (Qiagen, USA) including an on-column DNase digestion with the RNAse-free DNase set (Qiagen, USA) and quantified using Nabi microdigital spectrophotometer. cDNA synthesis was performed using the Verso cDNA synthesis kit (Thermo Scientific™) and stored at −80 °C until further use.

RT-PCR was conducted using qTOWER3G RT-PCR machine (Analytik Jena, Germany). Primer sequences selected for the study were: IL-17A: F-5′ TCACCCCGATTGTCCACCAT-3’/R-5′ GAGTTTAGTCCGAAATGAGGCTG-3’; IL-23: F- 5′-GCTTCAAAATCCTTCGCAG-3’/R-5′GATCTGAGTGCCATCCTTGAG-3’; Peptidyl-prolyl *cis*-trans isomerase (PPIA) (Housekeeping gene): F-5′-CCCACCGTGTTCTTCGACATT-3’/R-5′-GGACCCGTATGCTTTAGGATGA-3’. Amplification of cDNA was carried out by mixing cDNA with PowerUp™ SYBR Green Master Mix (Applied Biosystems) at the ratio of 1:10 and values were normalized against the endogenous control gene (PPIA). RT-PCR cycling conditions were set at initial denaturation: 94 °C for 5 min, followed by 45 cycles of denaturation 94 °C for 30 s; annealing: 60 °C for 30 s; extension: 72 °C for 45 s and final extension: 72 °C for 5 min. All the experiments were performed in triplicates and the quantification was performed by calculating the 2^−ΔΔCt^.

### 2.9 Enzyme-linked immunosorbent assay

Stored cell culture media from the LPS stimulated THP-1 cells, treated with DKV was used for the analysis of TNF-α and IL-6 using ELISA. Assays were performed following the manufacturer's protocol and absorbance was recorded at 450 nm using the Envision microplate reader (PerkinElmer, USA).

### 2.10 Statistical analysis

The data are expressed as Mean ± Standard Error of Mean (SEM) for each group. Statistical analysis was performed using GraphPad Prism version 7.03 (GraphPad Software, Inc., San Diego, CA, USA). A one-way analysis of variance (ANOVA) followed by Dunnett's multiple comparisons post-hoc test was used to calculate the statistical significance in-ear edema, biopsy weights, epidermal thickness, lesion scores, cytokine(s), and myeloperoxidase analysis. All analyses and comparisons were evaluated at a 95% level of confidence (p < 0.05).

## 3 Results

### 3.1 Phytochemical Profiling of DKV and DKO components

High-performance liquid chromatography (HPLC) analysis of the DKV and DKO using parameters of retention time (RT) and individual phytochemical standard curve showed DKV samples to be composed of 9.05 μg/mg Gallic acid (RT 6.3 min); 23.19 μg/mg Catechin (RT 11.4 min); 0.02 μg/mg Berberine (RT 17.6 min); and 0.0012 μg/mg Curcumin (RT 30.5 min) (Suppl. Table 2, Suppl. Fig. 1A and 1B). DKO samples were composed of 0.0029 μg/mg Gallic acid (RT 6.3 min); 0.0003 μg/mg Catechin (RT 11.4 min); 0.0056 μg/mg Berberine (RT 17.6 min); 0.002 μg/mg Phenol (RT 17.9 min); Benzoic acid (RT 19.6 min); and Curcumin (RT 30.5 min) (Suppl. Table 2, Suppl. Fig. 1C and 1D).

**Fig. 1:**
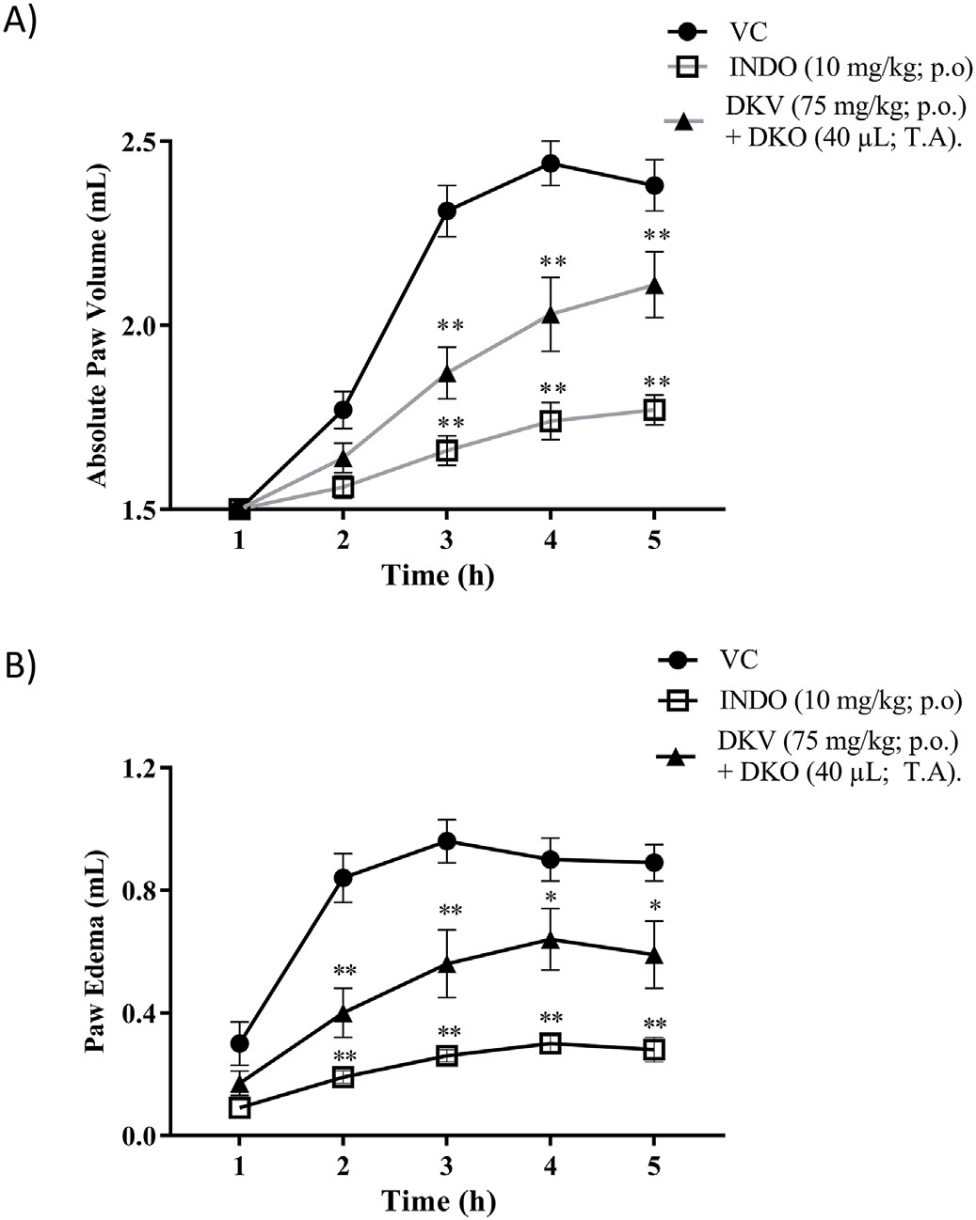
Effect of DKV-O treatment on λ-Carrageenan Stimulated Paw-Edema Rat Model. Treatment of λ-carrageenan stimulated Wistar rats with 75 mg/kg DKV (p.o.) and 40 μL DKO (T.A.), or with INDO (10 mg/kg) (p.o.) significantly reduced their A) absolute paw volume, and B) paw edema indicating anti-inflammatory efficacy. Statistical analysis of the treatments was performed using a one-way analysis of variance (ANOVA) followed by Newman-Keuls multiple comparison test. p-values ∗<0.05, ∗∗<0.01 (DC *versus* DKV-O treatment; DC *versus* INDO treated animals).

### 3.2 Anti-inflammatory effects of DKV-O in λ-carrageenan stimulated wistar rats

Plantar injection of Wistar rats with λ-carrageenan (0.1 ml of 1% solution in normal saline) stimulated an increase in the absolute paw volume and edema up to 5 h (Fig. 1A and B). Treatment of the λ-carrageenan stimulated rats with 75 mg/kg of DKV (p.o.) + 40 μl/paw of DKO (T.A.) significantly reduced the absolute paw-volume and edema (Fig. 1A and B). Treatment of the λ-carrageenan stimulated rats with positive control INDO (10 mg/kg) (p.o.) significantly reduced both the paw volume and edema compared to the vehicle control animal (Fig. 1A and B).

### 3.3 Anti-Ps-like-inflammation activity of DKV-O in TPA-Stimulated CD-1 mice

**Fig. 2:**
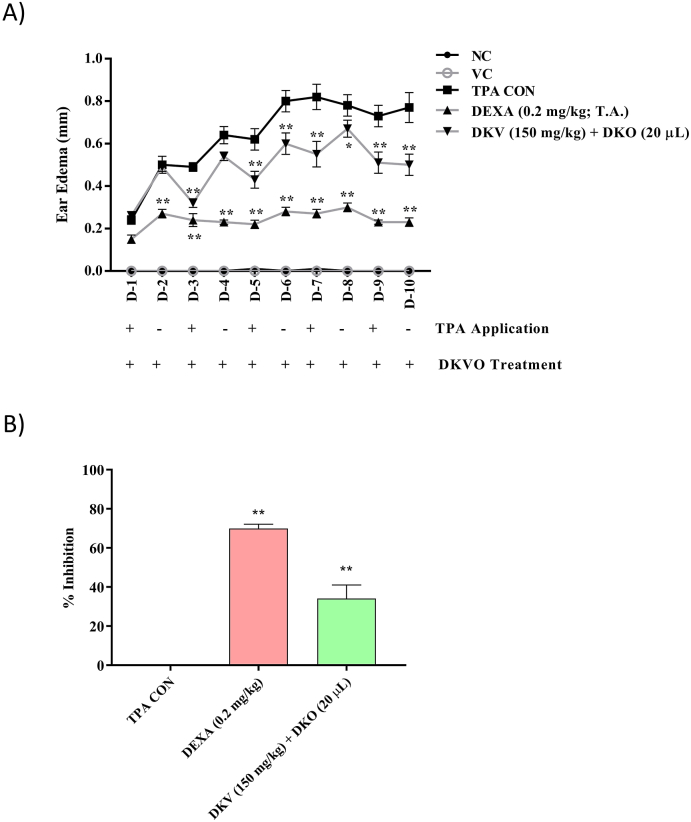
Effect of DKV-O Treatment on TPA-Stimulated Ear Edema in Mice. A) Treatment of the TPA-stimulated Ps-like inflamed ear (TPA CON) with DKV-O, or with DEXA (T.A.) significantly reduced ear edema. Vehicle control (VC) did not induce any change. B) Both, DKV-O, and DEXA induced significantly high inhibition in the ear stimulated with TPA alone (TPA CON). Statistical analysis was performed using a two-way analysis of variance (ANOVA) followed by Newman-Keuls multiple comparison test. P-values #<0.01 (Normal Control (NC) *versus* TPA CON); ∗<0.05, ∗∗<0.01 (TPA CON *versus* DKV-O co-treatment; TPA CON *versus* DEXA).

**Fig. 3:**
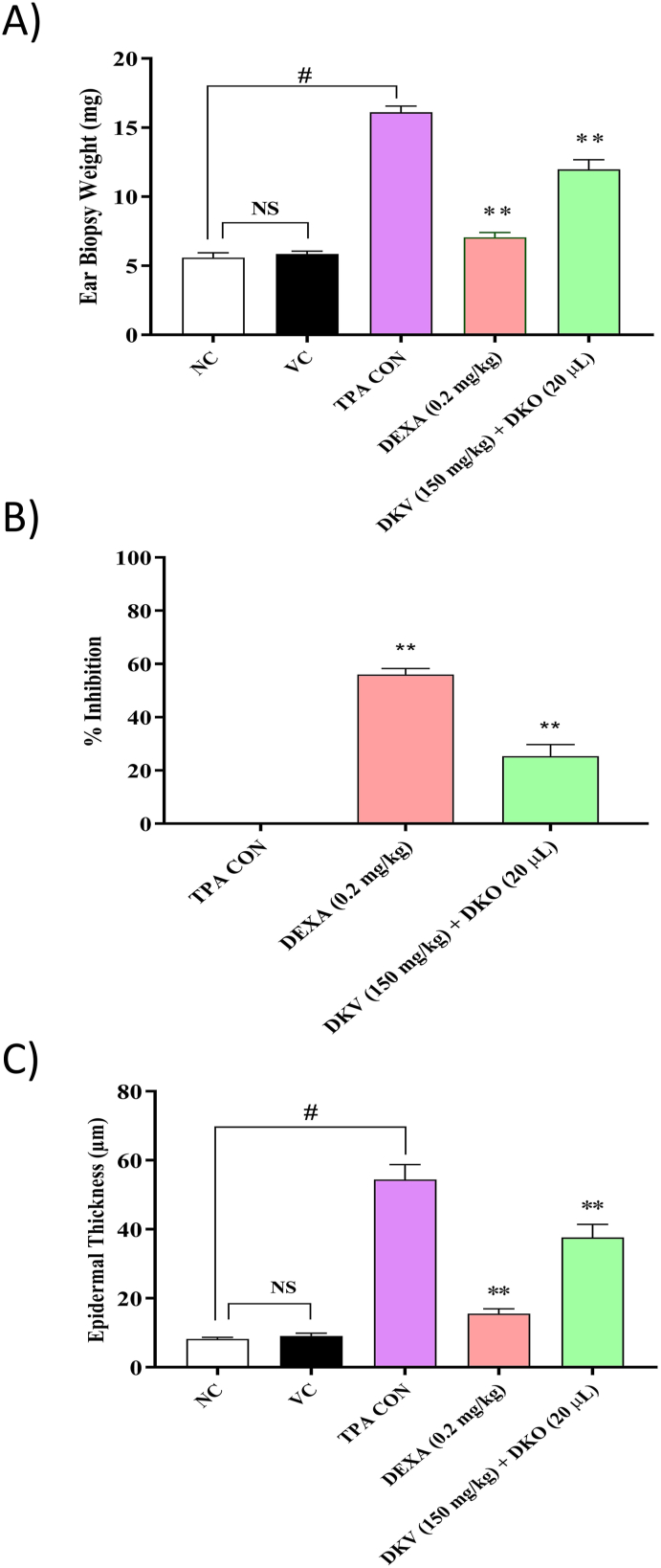
Histopathological Analysis of DKV-O Treatment on TPA-Stimulated Mice Ear Biopsy. A) Treatment of the TPA-stimulated Ps-like inflamed ear with DKV-O, or with DEXA (T.A.) significantly decreased the ear biopsy weight. B) Percent inhibition of TPA (TPA CON) stimulated Ps-like ear biopsy weight by DKV-O, or DEXA were found significantly high, and C) Treatment of the Ps-like inflamed ear with DKV-O, or DEXA significantly reduced the ear epidermal thickness. Vehicle Control (VC) alone did not cause any modification in the ear biopsy-weight or epidermal thickness. Statistical analysis was performed using the One-way analysis of variance (ANOVA) method followed by Dunnett's multiple comparison *t*-test. P-values #<0.01 (Normal Control (NC) *versus* TPA CON); ∗ <0.05, ∗∗ <0.01 (TPA CON *versus* DKV and DKO co-treatment; TPA CON *versus* DEXA).

Stimulation of the CD-1 mice right ear with TPA (2.5 μg/ear) induced Ps-like inflammation through an increase in ear edema, biopsy weight, and epidermal thickness (Fig. 2, Fig. 3). Treatment of the stimulated CD-1 mice with 150 mg/kg DKV (p.o.) and 20 μL DKO (T.A.) significantly (p-value <0.01) reduced the ear edema with an inhibition percentage of 34.2 ± 19.5% (Fig. 2A and B).

Treatment of TPA-stimulated ear with DKV-O also significantly (p-value <0.01) reduced the elevated ear biopsy-weight with an inhibition percentage of 25.3 ± 12.3% and the epidermal layer thickness (Fig. 3A–C). DEXA treatment of the stimulated ear also significantly (p-value <0.01) reduced the TPA-stimulated ear edema, biopsy weight and epidermal thickness (Fig. 2, Fig. 3).

### 3.4 Effect of DKV-O treatment on ear tissue histopathology in CD-1 mice

**Fig. 4:**
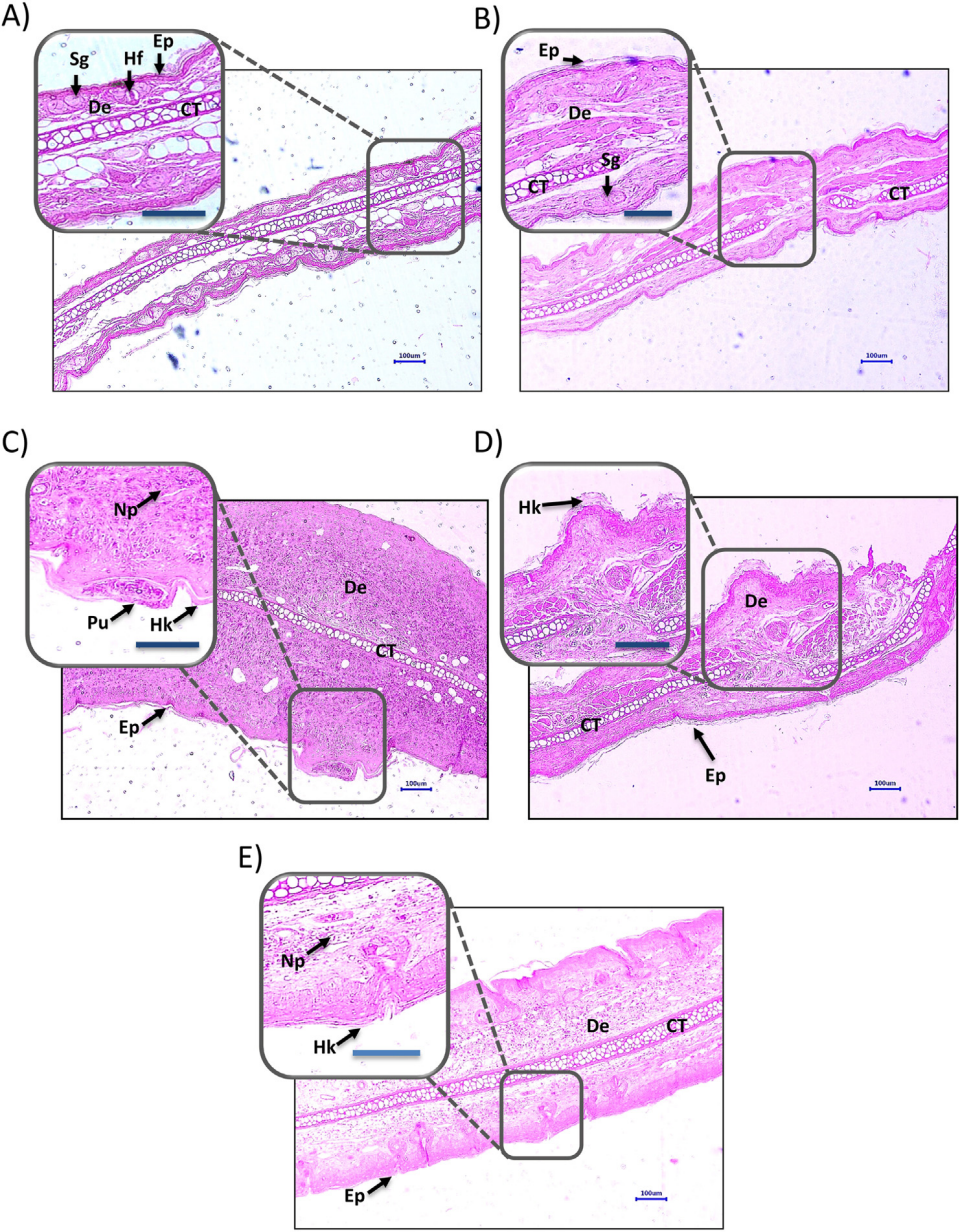
Histopathological Analysis of DKV-O Treatment on TPA-Stimulated Ear Ps in Mice. Histopathological analysis of the CD-1 mouse-ear dermal region showed A) Normal epidermis (Ep), dermis (De), sebaceous gland (Sg), cartilage (CT) and hair follicle (Hf) in the normal control animals; B) Normal Ep, De, Sg, CT in the vehicle control animals; C) Induction of hyperkeratosis (Hk) and hyperplastic Ep, pustule formation (Pu), and presence of neutrophils (Np) in dermis (De) region of TPA stimulated animal; D) Reduced hyperkeratosis (Hk) and hyperplastic Ep, and absence of neutrophils in the dermis (De) region of DEXA treated ear of TPA-stimulated animal and E) Reduced Hk and hyperplastic Ep, the reduced presence of neutrophils (Np) in the dermis (De) region of the DKV-O treated ear of TPA-stimulated animal.

TPA-stimulation of the right ear in CD-1 mice induced epidermal hyperkeratosis, hyperplasticity, pustule formation, and neutrophils influx in the dermis region (Fig. 4A–C). Treatment of the TPA-stimulated mice with DKV-O, or DEXA significantly (p-value <0.01) reduced the incidences of inflammatory lesion formation in the epithelial region, pustule formation, and an influx of the neutrophils (Fig. 4D and E).

**Fig. 5:**
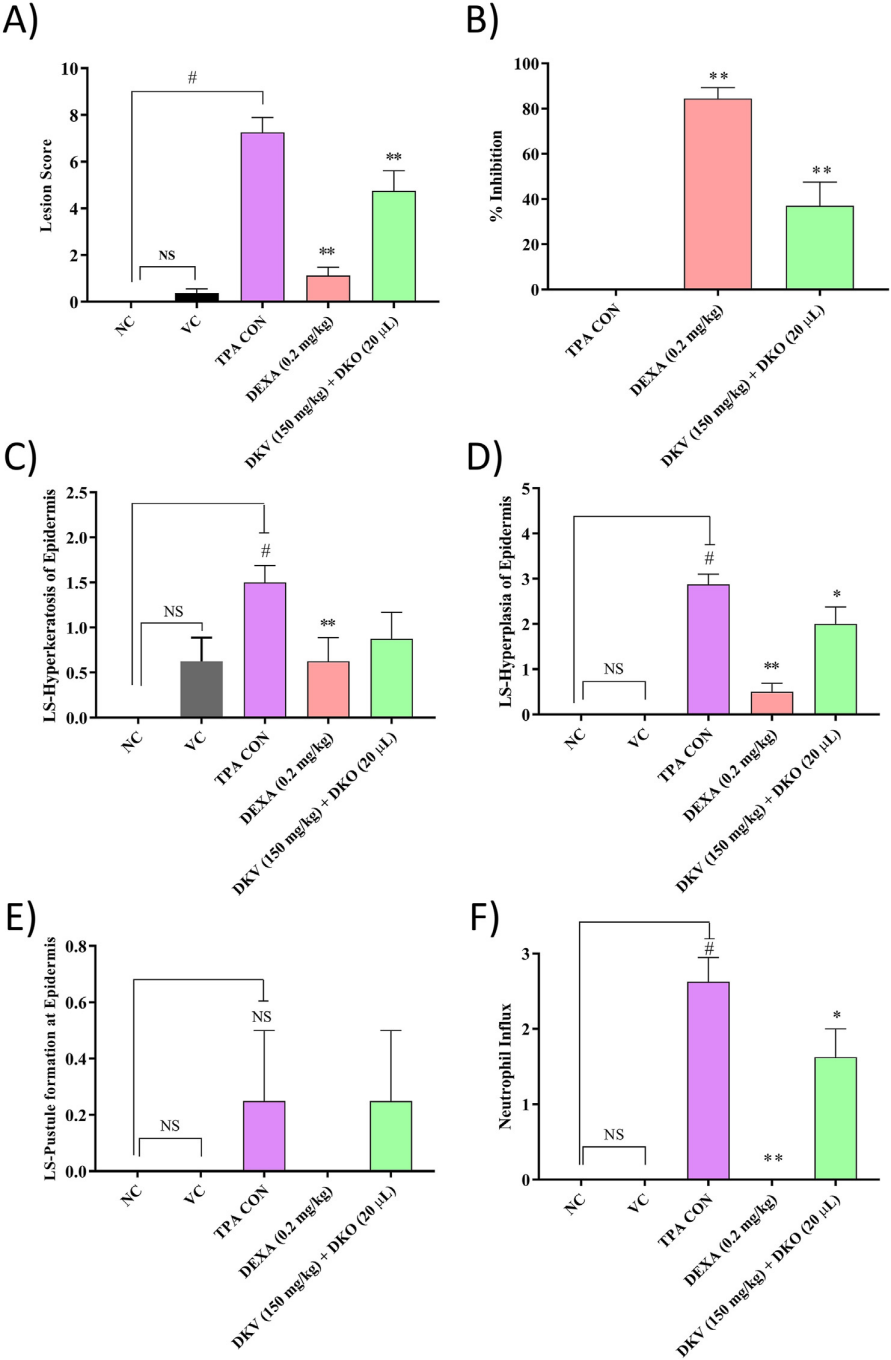
Inflammatory Lesion Scores in the DKV-O Treated and TPA-Stimulated Mice Ear. Treatment of the Ps-like inflamed ear with DKV-O, or with DEXA (T.A.) showed a reduction in TPA (TPA CON) stimulated- A) Total lesion score; B) Percentage inhibition of total lesion score in TPA CON *versus* DKV-O, or DEXA treated animals; C) Hyperkeratosis of the epidermis; D) Hyperplasia of the epidermis; E) Pustule formation in epidermis region; F) Neutrophil influx in dermis region. For statistical analysis, a one-way analysis of variance (ANOVA) followed by Dunnett's multiple comparison *t*-test was applied. P-value # <0.01 (Normal Control (NC) *versus* TPA CON). ∗ <0.05, ∗∗ <0.01 (TPA CON *versus* DKV and DKO treatments; TPA CON *versus* DEXA treatment).

Quantification of the observed histopathological effects through lesion score analysis showed DKV-O treatment to significantly (p-value <0.01) reduce the TPA-induced inflammatory lesions with a percent inhibition of 37.05 ± 10.52% (p-value <0.01) (Fig. 5A and B). Individually, DKV-O treatment ameliorated TPA-induced hyperkeratosis, and hyperplasia (p-value <0.05) in the epidermis, and neutrophil influx (p-value <0.05) in the dermal region of the stimulated ear of mice (Fig. 5C, D and 5F). No effect was observed on TPA-induced pustule formation from the DKV-O treatment (Fig. 5E). DEXA treatment also showed high anti-inflammatory efficacy through inhibition for all histopathological parameters observed having a percent inhibition of 84.47 ± 4.83% (p-value <0.01) (Fig. 5A).

### 3.5 *In-vitro* anti-inflammatory activity of DKV

**Fig. 6:**
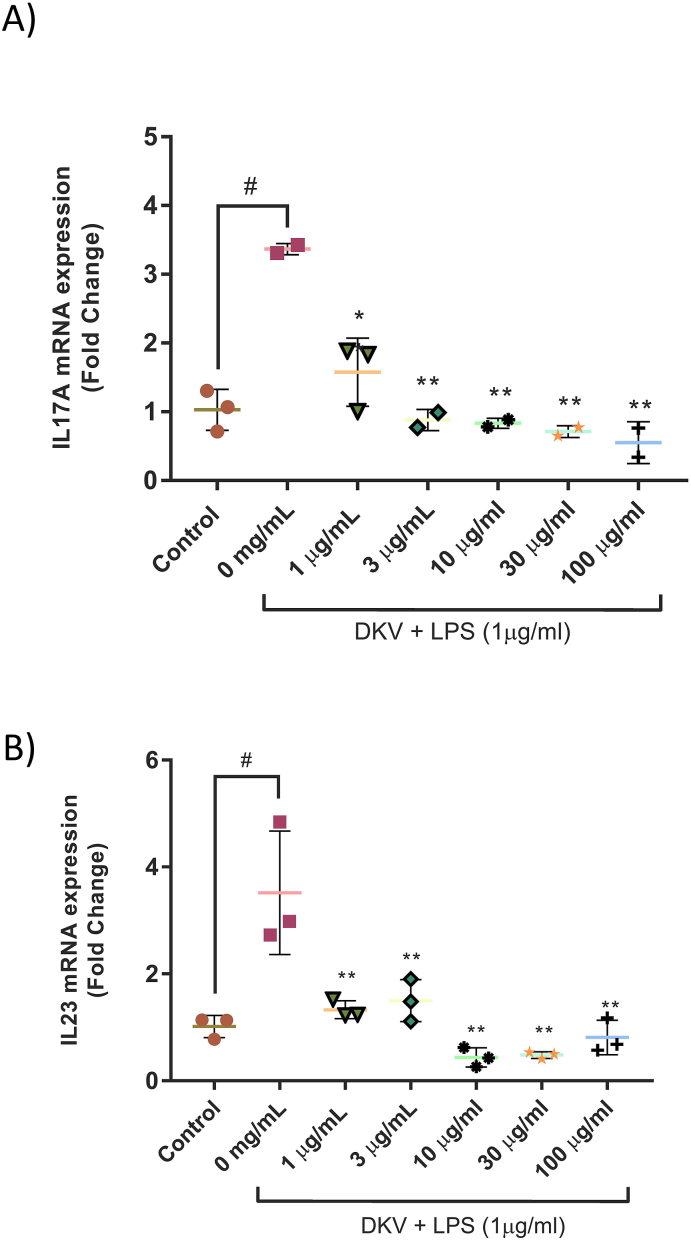
IL-17A and IL-23 mRNA Expression Changes in LPS-Stimulated THP-1 cells following DKV treatment: LPS (1 μg/ml) stimulation of the PMA-transformed THP-1 cells showed an increase in the mRNA expression levels of A) IL-17a, and B) IL-23 cytokine and their amelioration following treatment with DKV. Results are represented as Mean ± SEM (n = 3). For statistical analysis, one-way ANOVA followed by Dunnett's post-hoc test was performed. p-value # <0.001 (Control *versus* LPS only); p-value ∗∗ <0.01 (LPS only *versus* DKV treatments).

**Fig. 7:**
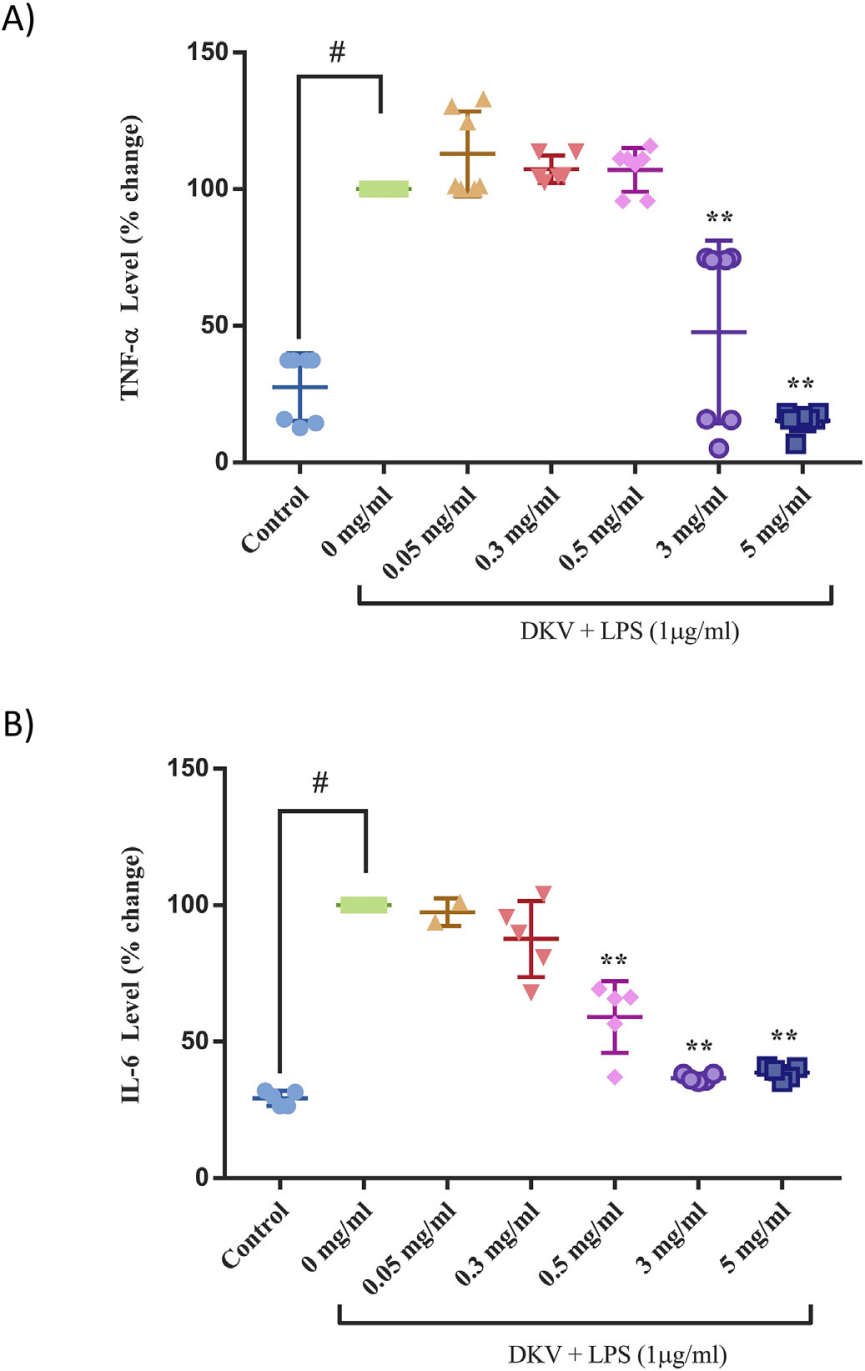
Release of TNF-α and IL-6 in LPS-Stimulated THP-1 cells following DKV treatment: LPS (1 μg/ml) stimulation of the PMA-transformed THP-1 cells leading to the release of A) TNF-α, and B) IL-6 cytokines and their amelioration following treatment with DKV. Inset showing percent inhibition. Results are represented as Mean ± SEM (n = 3). For statistical analysis, one-way ANOVA followed by Dunnett's post-hoc test was performed. p-value # <0.001 (Control *versus* LPS only); p-value ∗∗ <0.01 (LPS only *versus* DKV treatments).

LPS-stimulation of the PMA-transformed THP-1 cells showed an upregulation of pro-inflammatory cytokines- IL-17A, IL-23, TNF-α, and IL-6 (Fig. 6, Fig. 7). Pre-treatment of the THP-1 cells with varying concentrations of DKV showed a significant (p-value <0.01) reduction in the mRNA expression of IL-17A and IL-23 cytokines following LPS-stimulation (Fig. 6A and B). Similarly, DKV pre-treatment also reduced the LPS-stimulated release of TNF-α and IL-6 cytokines in the THP-1 cells (Fig. 2A and B). Statistically significant (p-value <0.01) reduction of TNF-α release from the LPS-stimulated THP-1 cells was only detectable at the DKV treatment concentrations of 3 mg/ml (47%) and 5 mg/ml (15%) (Fig. 7A). Similarly, a statistically significant (p-value <0.01) reduction in IL-6 cytokine release from the THP-1 cells was observed following pre-treatment with DKV at the concentrations 0.3–5 mg/ml (Fig. 7B).

Based on the obtained results, DKV-O treatment showed anti-inflammatory activity in Ps-like systemic inflammation through modulation of neutrophils influx, pro-inflammatory cytokine release and lesion formation.

## 4 Discussion

Skin is the outermost physical protective barrier of the body providing protection and maintaining physiological homeostasis. Initiation of Ps-like inflammation is represented by infiltration of the innate immune cells such as macrophages, neutrophils, TH17 and dendritic cells in the dermal region of the affected site. This is accompanied by the production of cytokines and chemokines, and proliferation of the keratinocytes leading to thickening of the epidermal region.^31^

Herbal formulations play an important role in the treatment of human diseases without any known side effects.^32^ Their therapeutic properties have been attributed to the presence of a wide variety of phytochemicals acting together in synergy and through a multicentric approach towards diseases.^32^^,^^33^ Herbal components of DKV such as *Phyllanthus emblica* L., *Curcuma longa* L. contain phytochemicals that have anti-inflammatory and antioxidant activities against skin inflammation.^34^, ^35^, ^36^ In our study, phytochemicals such as Gallic acid, Catechin, Berberine, Curcumin, Phenol, and Benzoic acid were found to be present in both DKV and DKO. Gallic acid can modulate the NADPH-oxidase activity in polymorphonuclear leukocytes associated with induction of oxidative stress through reactive oxygen species generation.^37^ Curcumin has been well-known for ages to have antioxidant and anti-inflammatory activity.^38^ Similarly, Catechin, Berberine, and Curcumin have been found to have anti-inflammatory and antioxidant abilities through modulation of inflammatory cells and mediators.^39^, ^40^, ^41^, ^42^, ^43^, ^44^, ^45^, ^46^

Macrophages play an important role in the pathogenicity of Ps through the release of pro-inflammatory cytokines and modulating the IL-23/IL-17 pathway.^6^ During the onset of Ps lesions, macrophages are present in large quantities and involved in the release of pro-inflammatory cytokines.^6^ In our study, LPS-stimulation of the PMA-transformed THP-1 cells showed an increase in the levels of IL-17A, IL-23, TNF-α and IL-6 that was ameliorated by DKV treatment. These soluble mediators are considered biomarkers of Ps disease as they process the inter-and intracellular signaling between the innate cells and affected dermal keratinocytes.^47^^,^^48^ IL-17 and IL-23 are produced by the innate and adaptive immune cells, and keratinocytes lead to the formation of epidermal hyperplasia. In our study, pro-inflammatory macrophage activity modulation by DKV indicated the anti-inflammatory contribution of the phytochemicals present in the herbal formulations.^49^^,^^50^

*In-vivo* efficacy of the DKV-O was observed in systemic acute inflammation models of λ-carrageenan stimulated Wistar rat paw edema and TPA-stimulated ear edema. The biphasic response of λ-carrageenan in stimulating rat paw-edema and TPA-modulated ear edema is mediated through the release of several pro-inflammatory soluble mediators, such as cytokine, chemokines and growth factors.^22^^,^^51^ In our study, the DKV-O combination efficiently reduced the λ-carrageenan and TPA stimulator-induced edema, hyperkeratosis, hyperplasia in the epidermal region, and inflammatory cell influx in the dermal region in the treated animals. TPA is known to induce Ps-like inflammation through the influx of inflammatory cells and modulation of IL-17 cytokine that eventually leads to keratinocyte growth promotion.^26^ Similarly, carrageenan is known to trigger the release of pro-inflammatory cytokines including IL-6, TNF-α and IL-17 inducing the inflammatory cascade.^52^ Hence, our observed anti-inflammatory activity of DKV-O can be correlated to the observed reduction in the inflammatory cell influx and development of inflammatory epidermal lesions.

Earlier studies on individual plant components used in the preparation of DKV and DKO such as *Cassia tora* L., *Berberis aristata* DC., *Curcuma longa* L., *Caesalpinia bonducella* (L.) Fleming, *Acacia catechu* (L.F.) Willd. and *Azadirachta indica* A. Juss. have shown amelioration of Ps-like inflammation.^53^, ^54^, ^55^, ^56^, ^57^, ^58^, ^59^, ^60^, ^61^, ^62^ Phytochemicals such as Berberine, and Curcumin present in DKV-O have individually shown efficacy to reduce carrageenan and TPA stimulated inflammation in animal models through the modulation of the cytokines, nitric oxides, cyclooxygenase and prostaglandins.^40^^,^^63^, ^64^, ^65^, ^66^ Furthermore, both Berberine and Curcumin have been observed to modulated the IL-17 and IL-23 expression in the immune cells.^67^, ^68^, ^69^

## 5 Conclusion

Taken together, DKV showed good efficacy in the modulation of pro-inflammatory cytokines in LPS-stimulated macrophages. In the λ-carrageenan and TPA-stimulated acute inflammation animal models, DKV-O treatment reduced paw and ear edema, epidermal thickness, and Ps-like inflammatory lesions. Phytochemicals present in the DKV and DKO play an important role through a possible multicentric synergistic effect in the modulation of Ps-like inflammatory response that needs further exploration. Hence, combined treatment of Divya-Kayakalp-Vati and Divya-Kayakalp-Oil can be explored further for the treatment of inflammatory dermal diseases like Psoriasis.

## Author contributions

AB provided a broad direction for the study, identified the test formulation, generated resources, and gave final approval for the manuscript. SS conducted the *in-vivo* study, analyzed the data, and performed report writing. KJ assisted in animal handling and in performing *in-vivo* studies. RS and KB performed *in-vitro* experiments and RT-PCR analysis. SV and PN conducted analytical experiments. KB performed data curing and wrote the manuscript. AV supervised overall research project planning, generated resources, and reviewed and finally approved the manuscript.

## Funding

This research work was funded internally by Patanjali Research Foundation Trust, Haridwar, India.

## Declaration of competing interest

The test article was sourced from Divya Pharmacy, Haridwar, Uttarakhand, India. Acharya Balkrishna is an honorary trustee in Divya Yog Mandir Trust, Haridwar, India. In addition, he holds an honorary managerial position in Patanjali Ayurved Ltd., Haridwar, India. Besides, providing the test article, Divya Pharmacy was not involved in any aspect of this study. All other authors have no conflict of interest to declare.
