# Supplementary material for: Modulation of psoriatic-like skin inflammation by traditional Indian medicine Divya-Kayakalp-Vati and Oil through attenuation of pro-inflammatory cytokines
Source: J Tradit Complement Med. 2021 Sep 20;12(4):335–44. doi: 10.1016/j.jtcme.2021.09.003 (PMC9210137; doi:10.1016/j.jtcme.2021.09.003)
Supplement: Multimedia component 1 [file mmc1.docx]

**SUPPLEMENTARY INFORMATION**

**Modulation of Psoriatic-like Skin Inflammation by Traditional Indian Medicine Divya-Kayakalp-Vati and Oil Through Attenuation of Pro-Inflammatory Cytokines**

Acharya Balkrishna^1,2^, Sachin Sakat^1^, Kheemraj Joshi^1^, Rani Singh^1^, Sudeep Verma^1^, Pardeep Nain^1^, Kunal Bhattacharya^1^, Anurag Varshney^1,2*^

**Author Affiliations:**

1. Drug Discovery and Development Division, Patanjali Research Institute, NH-58, Haridwar-249405, Uttarakhand, India.
2. Department of Allied and Applied Sciences, University of Patanjali, Patanjali YogPeeth, Haridwar, India

***Corresponding Author:**

Dr. Anurag Varshney

Drug Discovery and Development,

Patanjali Research Institute,

NH-58, Haridwar-249405 (Uttarakhand)

India

T: [+91 1334-244107 x7458](tel:+911334244107,7458) F: [+91 1334 244805](tel:+911334244805)

Email: [anurag@prft.co.in](mailto:anurag@prft.co.in)

**SUPPLEMENTARY TABLE 1**: Herbal Components of Divya-Kayakalp-Vati as mentioned in the ancient Indian medicinal texts [*Bhav Prakash Nighantu* (B.P.N.) and *Ayurved Saar Sangrah* (A.S.S.)].

| **S.No.** | **Vernacular Name** | **Scientific Name** | **Part Used** | **Quantity (mg/tablet)** | **Ref. Book: Page Number** |
| --- | --- | --- | --- | --- | --- |
| 1 | Panvad | *Cassia tora* L. | Seed | 18.75 | B.P.N.: 125-126 |
| 2 | Haldi | *Curcuma longa* L. | Rhizome | 18.75 | B.P.N.: 114 |
| 3 | Daru Haldi | *Berberis aristata* DC. | Rhizome | 37.50 | B.P.N.: 119 |
| 4 | Khair | *Acacia catechu* (L.F.) Willd. | Stem bark | 25.00 | B.P.N.: 525-526 |
| 5 | Karanj | *Caesalpinia bonducella* (L.) Fleming | Seed | 6.25 | B.P.N.: 352 |
| 6 | Neem | *Azadirachta indica* A. Juss. | Leaf | 25.00 | B.P.N.: 329-331 |
| 7 | Amla | *Emblica officinalis* Gaertn. | Fruit rind | 37.50 | B.P.N.: 10-12 |
| 8 | Manjishta | *Rubia cordifolia* L. | Root | 6.25 | B.P.N.: 110-111 |
| 9 | Giloy | *Tinospora cordifolia* (Willd.) Hook.f. & Thomson | Stem | 18.75 | B.P.N.: 269-271 |
| 10 | Chirayata | *Swertia chirata* Buch.-Ham. (ex Wall) | Whole Plant | 18.75 | B.P.N.: 73-74 |
| 11 | Kutaki | *Picrorhiza kurroa* Royale ex. Benth. | Root | 6.25 | B.P.N.: 70-71 |
| 12 | Dronpushapi | *Leucas cephalotes* (Roth) Spreng. | Extract | 6.25 | B.P.N.: 463-464 |
| 13 | Satyanashi | *Argemone Mexicana* L.) | Extract | 12.50 | B.P.N.: 96-98 |
| 14 | Ras manikya | Red Sapphire ash | -- | 6.25 | A.S.S.: 383 |

**SUPPLEMENTARY TABLE 2:** Phytochemical analysis of Divya-Kayakalp-Vati (DKV) and Divya-Kaykalp-Oil (DKO) using HPLC analysis (Refer to Figure 1). (RSD%: Relative Standard Deviation Percent).

|  | **Analyte** | **Content of phytochemicals**  **(µg/mg)** | **Retention Time**  **(min)** | **Intra day**  **(n =6)**  **RSD %** | **Inter day**  **(n =6)**  **RSD %** |
| --- | --- | --- | --- | --- | --- |
| **DKV** | Gallic Acid | 9.05 | 6.341 | 1.64 | 2.08 |
|  | Catechin | 23.19 | 11.399 | 1.70 | 2.71 |
|  | Berberine | 0.0222 | 17.590 | 1.68 | 1.76 |
|  | Curcumin | 0.00125 | 30.542 | 1.94 | 1.95 |
| **DKO** | Gallic Acid | 0.00294 | 6.344 | 4.81 | 2.92 |
|  | Catechin | 0.000326 | 11.407 | 2.84 | 2.45 |
|  | Berberine | 0.00561 | 17.371 | 3.33 | 2.35 |
|  | Curcumin | 0.00138 | 30.543 | 2.37 | 2.49 |
|  | Phenol | 0.00268 | 17.969 | 1.90 | 2.13 |

A

B

C

D

**SUPPLEMENTARY FIGURE 1: HPLC Analysis of Divya-Kayakalp-Vati (DKV) and Divya-Kayakalp-Oil (DKO):** Phytochemicals identified using HPLC in DKV based on their retention time (RT) and standard mix were **A)** Gallic acid (RT 6.3 min), Catechin (RT 11.4 min) measured at 275 nm, and **B)** Berberine (RT 17.6 min) and Curcumin (RT 30.5 min) measured at 425 nm. In DKO phytochemicals identified were **C)** Gallic acid (RT 6.3 min); Catechin (RT 11.4 min); Berberine (RT 17.3 min); Phenol (RT 17.9 min); and Benzoic acid (RT 19.6 min) measured at 275 nm, and **D)** Curcumin (RT 30.5 min) measured at 425 nm. Experiments were repeated six times to determine the relative standard deviation (RSD) within <2.5% between inter- and intra-days’ measurements (See Suppl. Table 2).
